# *Porphyromonas gingivalis*-Associated Modulation of β-Catenin Signaling in Oral Squamous Cell Carcinoma: Molecular Perspectives from Periodontal Dysbiosis

**DOI:** 10.3390/molecules31050901

**Published:** 2026-03-09

**Authors:** Nada Tawfig Hashim, Rasha Babiker, Riham Mohammed, Mariam Elsheikh, Vivek Padmanabhan, Md Sofiqul Islam, Malaz Gesm Elseed, Nallan C. S. K. Chaitanya, Bogahawatte Samarakoon Mudiyanselage Samadarani Siriwardena, Muhammed Mustahsen Rahman, Bakri Gobara Gismalla

**Affiliations:** 1 Department of Periodontics, RAK College of Dental Sciences, RAK Medical and Health Sciences University, Ras-AlKhaimah 12973, United Arab Emirates; mustahsen@rakmhsu.ac.ae; 2 Department of Oral Rehabilitation, Faculty of Dentistry, University of Khartoum, Khartoum 11115, Sudan; bakrigobara10@gmail.com; 3 Department of Physiology, RAK College of Medical Sciences, RAK Medical and Health Sciences University, Ras-AlKhaimah 11172, United Arab Emirates; rashababiker@rakmhsu.ac.ae; 4 Department of Oral Surgery, RAK College of Dental Sciences, RAK Medical and Health Sciences University, Ras-AlKhaimah 12973, United Arab Emirates; riham.abdelraouf@rakmhsu.ac.ae; 5 Department of Oral & Maxillofacial Surgery, Faculty of Dentistry, University of Khartoum, Khartoum 11115, Sudan; mariam.elhadi@oofk.edu; 6 Department of Pediatric and Preventive Dentistry, RAK College of Dental Sciences, RAK Medical and Health Sciences University, Ras-AlKhaimah 12973, United Arab Emirates; vivek.padmanabhan@rakmhsu.ac.ae; 7 Department of Operative Dentistry, RAK College of Dental Sciences, RAK Medical and Health Sciences University, Ras-AlKhaimah 12973, United Arab Emirates; sofiqul.islam@rakmhsu.ac.ae; 8 Department of Oral Pathology, Faculty of Dentistry, University of Khartoum, Khartoum 11115, Sudan; malaz.elseed@oofk.edu; 9 Department of Oral Medicine and Radiology, RAK College of Dental Sciences, RAK Medical and Health Sciences University, Ras-AlKhaimah 12973, United Arab Emirates; krishna.chytanya@rakmhsu.ac.ae; 10 Department of Oral Pathology, RAK College of Dental Sciences, RAK Medical and Health Sciences University, Ras-AlKhaimah 12973, United Arab Emirates; samarakon@rakmhsu.ac.ae

**Keywords:** *P. gingivalis*, β-catenin, Wnt signalling, oral squamous cell carcinoma, periodontitis, gingipains, epithelial–mesenchymal transition

## Abstract

Periodontal disease and oral squamous cell carcinoma (OSCC) are highly prevalent conditions that contribute substantially to global morbidity, as documented by recent Global Burden of Disease analyses. The growing epidemiologic and experimental literature has prompted interest in potential links between chronic periodontal dysbiosis—particularly infection with *Porphyromonas gingivalis*—and molecular pathways involved in oral carcinogenesis, including β-catenin signaling. This narrative review synthesizes epidemiologic, clinical, and experimental studies examining associations between periodontal disease, *P. gingivalis*, and OSCC, with focused evaluation of β-catenin as a context-dependent signaling component within broader inflammatory and metabolic networks. Population-based studies report heterogeneous associations between periodontitis and OSCC that are frequently confounded by tobacco use, alcohol consumption, and socioeconomic factors, supporting correlation rather than causal inference. Experimental investigations in vitro and in vivo demonstrate that *P. gingivalis* can influence β-catenin availability and transcriptional activity through noncanonical mechanisms, including junctional disruption, proteolytic interference with regulatory complexes, and interaction with inflammatory, immune, and metabolic pathways. However, these findings derive largely from simplified model systems and should be interpreted as biologically plausible rather than definitive for human disease. Rather than acting as a dominant oncogenic driver, β-catenin appears to function as an integrative signaling node within a complex network shaped by chronic microbial and inflammatory stress. The principal contribution of this review lies in critically integrating dispersed evidence across study types while explicitly distinguishing association, mechanistic plausibility, and causality. Future longitudinal human studies and mechanistically informed experimental models are required to clarify whether modulation of periodontal dysbiosis or associated signaling pathways has relevance for OSCC risk assessment or prevention.

## 1. Introduction

The human oral cavity harbors one of the most diverse microbial ecosystems in the body, second only to the gastrointestinal tract, with more than 700 identified bacterial species forming complex multispecies biofilms that contribute to oral and systemic health [1]. Under conditions of dysbiosis, however, this microbial community is implicated in the pathogenesis of chronic inflammatory diseases, most notably periodontitis [2]. Globally, periodontal disease represents the most prevalent inflammatory condition, affecting more than 1.1 billion individuals, and successive cycles of the Global Burden of Disease (GBD) Study consistently rank severe periodontitis among the top ten causes of disability-adjusted life years worldwide [3,4]. Its increasing prevalence reflects ageing populations, shared behavioral risk factors, and persistent disparities in access to preventive oral healthcare across regions [4].

Oral squamous cell carcinoma (OSCC) likewise constitutes a major and growing global health challenge. Recent GBD estimates indicate that OSCC accounts for more than 377,000 new diagnoses and over 177,000 deaths annually, with substantial disability attributable to late-stage presentation and limited therapeutic responsiveness [5,6]. Although tobacco use, alcohol consumption, and betel quid chewing remain the dominant etiological factors, these exposures do not fully account for the observed incidence of OSCC, particularly among non-smoking and non-drinking populations, suggesting the potential involvement of additional biological and environmental modifiers [7,8].

Chronic oral inflammation and microbial dysbiosis, particularly periodontitis, have therefore emerged as plausible correlates of oral carcinogenesis [9]. Epidemiological studies have reported associations between clinical indicators of periodontal disease—including attachment loss, bone loss, and tooth loss—and OSCC risk [10,11,12]. However, these associations are heterogeneous and frequently attenuated after adjustment for smoking, alcohol use, and socioeconomic factors, and residual confounding remains a significant limitation. Moreover, periodontal disease may reflect cumulative lifestyle, inflammation, or healthcare access rather than an independent causal factor. These considerations underscore the need for cautious interpretation of epidemiologic findings and clear distinction between association and causation.

Among periodontal pathogens, *Porphyromonas gingivalis* (*P. gingivalis*) has received considerable attention in experimental research due to its ability to invade gingival epithelial cells, evade immune surveillance, remodel extracellular matrix components, and modulate host inflammatory responses [13,14]. This keystone pathogen expresses multiple virulence factors—including gingipains, atypical lipopolysaccharide, fimbriae, and outer membrane vesicles—that enable interaction with host signaling pathways [13]. Experimental studies have suggested that *P. gingivalis* can influence oncogenic signaling cascades relevant to epithelial transformation under controlled conditions [15].

Among the diverse host signaling pathways influenced by *P. gingivalis*, β-catenin represents a particularly informative mechanistic focal point due to its dual structural and transcriptional functions in epithelial biology. Unlike signaling cascades that operate primarily through downstream transcriptional modulation, β-catenin serves both as a core component of adherens junctions, regulating epithelial barrier integrity, and as a transcriptional co-activator governing genes involved in proliferation, differentiation, and epithelial plasticity [16]. This dual role uniquely positions β-catenin at the interface between microbial-induced disruption of epithelial junctional architecture and downstream transcriptional programs relevant to carcinogenesis. Furthermore, β-catenin signaling functions as a convergence point for multiple pathways modulated by *P. gingivalis*, including inflammatory signaling, protease-mediated junctional remodeling, metabolic reprogramming, and epithelial–mesenchymal transition [16]. Experimental evidence demonstrates that microbial perturbation of epithelial adhesion and inflammatory homeostasis can directly alter β-catenin stability and transcriptional activity, providing a mechanistically coherent link between periodontal dysbiosis and epithelial signaling alterations [17]. For these reasons, β-catenin provides a biologically grounded and integrative framework through which to examine microbial modulation of epithelial signaling without implying that it functions in isolation or as a universal driver of malignant transformation.

In this context, the Wnt/β-catenin signaling pathway has been highlighted as a potential mechanistic intersection. β-Catenin is a central regulator of epithelial proliferation, adhesion, survival, and differentiation, and its dysregulation is frequently observed in OSCC [15,18]. Experimental models indicate that *P. gingivalis* may modulate β-catenin signaling through noncanonical mechanisms, including disruption of E-cadherin-mediated adhesion, inflammatory cytokine-associated stabilization, and proteolytic interference with components of the β-catenin destruction complex [19,20]. Importantly, these findings are derived predominantly from in vitro systems and animal models, and their relevance to human OSCC remains to be fully established.

Given the parallel global burden of periodontitis and OSCC documented by recent GBD analyses [3,6], a critical appraisal of the proposed *P. gingivalis*–β-catenin–OSCC axis is timely. Rather than assuming causality, it is essential to evaluate the strength, limitations, and contextual relevance of existing epidemiologic and experimental evidence. Accordingly, this review aims to synthesize current data on *P. gingivalis*-associated modulation of β-catenin signaling within a balanced framework, distinguishing established observations from speculative mechanisms and identifying key gaps that warrant further investigation [21,22,23].

## 2. Methodological Approach and Scope of the Review

This article was designed as a narrative, hypothesis-driven review rather than a systematic review or meta-analysis. A structured literature search was conducted to identify relevant studies examining the relationships between periodontal disease, *P. gingivalis*, β-catenin signaling, and oral squamous cell carcinoma (OSCC). Electronic searches were performed in PubMed/MEDLINE, Web of Science, and Scopus databases covering the period from January 2000 to March 2025, selected to capture contemporary molecular and epidemiologic evidence while including foundational mechanistic studies.

The search strategy employed combinations of Medical Subject Headings (MeSH) and free-text keywords, including: “*P. gingivalis*,” “periodontitis,” “oral squamous cell carcinoma,” “oral cancer,” “β-catenin,” “Wnt signaling,” “gingipains,” “epithelial–mesenchymal transition,” “microbial dysbiosis,” and “oral carcinogenesis.” Boolean operators (AND, OR) were used to refine search specificity. Reference lists of relevant primary articles and review papers were manually screened to identify additional pertinent studies not captured through database searches.

Studies were considered eligible for inclusion if they met the following criteria: (1) peer-reviewed original research articles or high-quality reviews; (2) investigation of molecular, cellular, microbiological, epidemiologic, or experimental relationships involving *P. gingivalis*, periodontitis, β-catenin signaling, or OSCC; and (3) direct relevance to host–microbe signaling interactions or oral epithelial carcinogenesis. Studies were excluded if they lacked mechanistic relevance, focused on unrelated malignancies without oral epithelial context, or provided insufficient methodological detail to support interpretation.

Study selection and evaluation were performed independently by two authors with expertise in periodontology and oral molecular biology. Discrepancies in study relevance or interpretation were resolved through discussion and consensus to ensure balanced representation of the evidence. Particular attention was given to inclusion of studies presenting divergent or conflicting findings, which were critically examined within the manuscript to distinguish between association, mechanistic plausibility, and causal inference.

Given the narrative scope of this review, formal risk-of-bias assessment and quantitative synthesis were not performed. Instead, emphasis was placed on integrating representative and methodologically informative studies across epidemiologic and experimental domains, while explicitly acknowledging limitations, heterogeneity, and areas of uncertainty within the current evidence base. This approach was intended to provide a biologically grounded and critically balanced synthesis of the proposed *P. gingivalis*–β-catenin–OSCC signaling axis.

## 3. Epidemiologic and Clinical Associations Between Periodontitis, *P. gingivalis*, and OSCC

A growing body of research has examined potential associations between periodontitis, periodontal pathogens, and oral squamous cell carcinoma (OSCC) using epidemiologic, serologic, microbiological, histopathological, and experimental approaches. Collectively, these studies suggest a possible relationship between chronic periodontal disease and OSCC; however, the strength, consistency, and interpretation of these findings vary substantially across study designs and populations. The principal evidence across these different study types is summarized in (Table 1).

Across case–control and cohort studies, clinical indicators of periodontal disease—including clinical attachment loss, radiographic bone loss, and cumulative tooth loss—have been associated with OSCC risk [24,25,26]. While several analyses report persistence of these associations after adjustment for tobacco and alcohol exposure, effect sizes are heterogeneous, and residual confounding by socioeconomic status, healthcare access, nutrition, and cumulative inflammatory burden cannot be excluded. Importantly, periodontal status is frequently assessed cross-sectionally or retrospectively, limiting inference regarding temporality and raising the possibility that periodontal disease may act as a marker of shared risk factors rather than an independent causal determinant.

Serological investigations provide complementary but indirect evidence. Elevated serum titers of *P. gingivalis*-specific antibodies have been reported in OSCC patients compared with controls in some populations [27]. These findings are consistent with prior or chronic exposure to the organism but do not distinguish between active infection, historical colonization, or secondary immune responses to tumor-associated dysbiosis. Assay variability and population differences further contribute to inter-study heterogeneity.

Microbiological studies using PCR-based detection and sequencing approaches have identified *P. gingivalis* DNA more frequently in saliva, tumor-adjacent mucosa, and resected OSCC tissues than in control samples [27]. While these observations suggest enrichment of periodontal pathogens within OSCC-associated oral environments, detection of bacterial DNA does not establish viability, metabolic activity, or functional involvement in carcinogenesis. Moreover, tumor-associated ecological shifts may favor secondary colonization by anaerobic species, complicating causal interpretation.

Histopathological analyses have provided spatial context by localizing *P. gingivalis* within OSCC tissues, often at the invasive tumor front or within tumor-associated epithelium [28]. Co-localization with other dysbiotic taxa, including *Fusobacterium nucleatum*, has been reported, raising the possibility of polymicrobial interactions. However, spatial proximity alone does not establish mechanistic contribution, and such findings may reflect tumor-associated niche selection rather than pathogen-driven transformation.

Experimental carcinogenesis models offer additional insight but must be interpreted cautiously. In the 4-nitroquinoline-1-oxide (4NQO) mouse model, chronic oral exposure to *P. gingivalis*, alone or in combination with *F. nucleatum*, has been shown to increase tumor burden and accelerate dysplastic progression compared with carcinogen exposure alone [29]. These studies demonstrate that periodontal pathogens can modulate tumor-associated pathways under controlled conditions; however, the requirement for chemical carcinogen exposure and species-specific immune and microbial differences limit direct extrapolation to human OSCC.

Taken together, existing epidemiologic and experimental data support an association between periodontal disease, *P. gingivalis*, and OSCC-related biological processes, but they do not establish causality. The heterogeneity of findings, methodological limitations, and potential for confounding underscore the need to distinguish correlation from mechanistic plausibility when interpreting this body of evidence. Accordingly, subsequent sections focus on experimental insights into host–pathogen interactions while maintaining a clear separation between established observations and speculative mechanisms.

## 4. The Wnt/β-Catenin Pathway in Oral Epithelium and Carcinogenesis

In the canonical Wnt signaling pathway, β-catenin levels are tightly regulated by a cytoplasmic destruction complex composed of Axin, APC, GSK-3β, and CK1 [34]. In the absence of Wnt ligand stimulation, β-catenin is phosphorylated, ubiquitinated, and targeted for proteasomal degradation [34]. Upon binding of Wnt ligands to Frizzled and LRP receptors, the destruction complex is inhibited, allowing β-catenin to accumulate in the cytoplasm and translocate to the nucleus, where it associates with TCF/LEF transcription factors to regulate gene expression [34,35]. Target genes include regulators of proliferation, differentiation, and epithelial remodeling, such as c-MYC, CCND1, AXIN2, and MMP7.

In oral epithelial dysplasia and OSCC, aberrant β-catenin localization and activity have been reported in a subset of cases and are variably associated with histological grade, invasive behavior, and clinical outcome [36]. Mechanisms contributing to β-catenin dysregulation include altered Wnt ligand expression, loss of E-cadherin-mediated cell–cell adhesion, and epigenetic silencing of negative regulators of the pathway. Importantly, β-catenin activation is neither universal nor uniform across OSCC, reflecting the molecular heterogeneity of the disease and the contribution of multiple oncogenic signaling networks.

**Figure 1:**
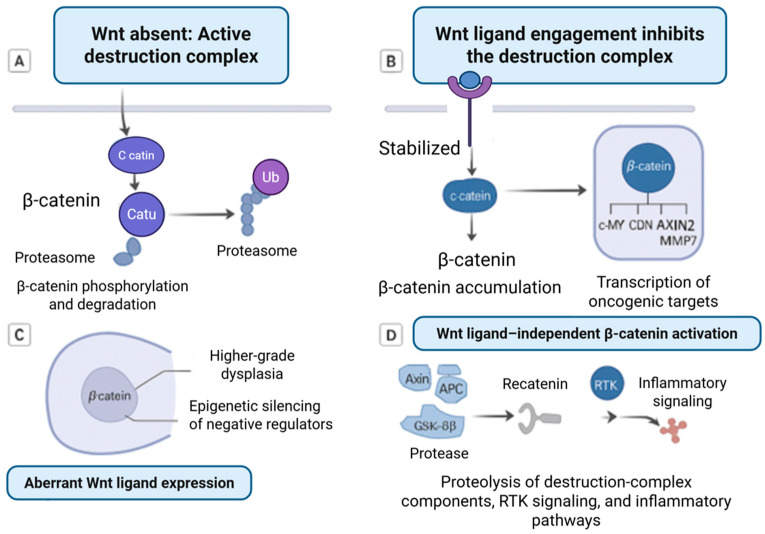
Regulation of β-catenin signaling through canonical and non-canonical mechanisms. (**A**) Active destruction complex in the absence of Wnt ligands. β-catenin is phosphorylated by the destruction complex (Axin, APC, GSK-3β, and CK1), leading to ubiquitination and proteasomal degradation, thereby preventing nuclear accumulation. (**B**) Wnt ligand engagement inhibits the destruction complex. Binding of Wnt ligands stabilizes β-catenin, allowing cytoplasmic accumulation and nuclear translocation, where it interacts with TCF/LEF transcription factors to activate oncogenic target genes such as c-MYC, Cyclin D1, and AXIN2. (**C**) Aberrant Wnt ligand expression. Dysregulated or excessive Wnt signaling promotes sustained β-catenin stabilization and nuclear signaling, contributing to transcriptional activation of oncogenic pathways and epigenetic silencing of negative regulators. (**D**) Wnt-independent β-catenin activation. β-catenin signaling may also be activated independently of Wnt ligands through alternative pathways, including receptor tyrosine kinase (RTK) signaling, inflammatory mediators, or disruption of destruction complex components, leading to increased β-catenin transcriptional activity. Created with BioRender.com.

Beyond canonical Wnt ligand-dependent activation, β-catenin can be influenced by noncanonical mechanisms independent of Wnt signaling, including disruption of the destruction complex, cross-talk with receptor tyrosine kinases, and inflammatory signaling cascades [31,37]. These alternative modes of regulation highlight how β-catenin may function as an integrative signaling node rather than a primary oncogenic driver. Within this framework, β-catenin dysregulation represents one of several context-dependent pathways implicated in oral carcinogenesis, providing a conceptual basis for examining how external factors—including microbial and inflammatory stimuli—may modulate epithelial signaling without implying pathway dominance or causality (Figure 1).

## 5. Experimental Evidence That *P. gingivalis* Activates β-Catenin Signalling

### 5.1. Noncanonical β-Catenin Activation by Gingipains

**Figure 2:**
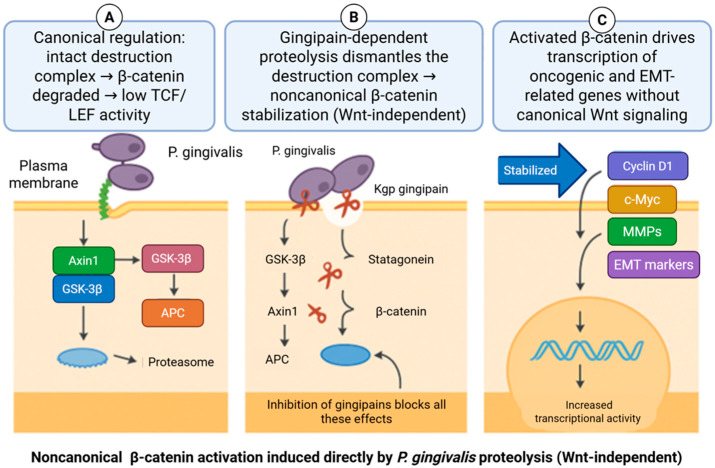
Noncanonical β-catenin activation induced by *Porphyromonas gingivalis*. (**A**) Under canonical conditions, β-catenin is phosphorylated by the APC/Axin/GSK-3β destruction complex and degraded, preventing transcriptional activity. (**B**) *P. gingivalis* gingipains proteolytically disrupt components of the destruction complex in a Wnt-independent manner, leading to β-catenin stabilization. (**C**) Stabilized β-catenin translocates to the nucleus and drives expression of oncogenic and EMT-related targets, including *Cyclin D1*, *c-MYC*, MMPs, and EMT markers, promoting transcriptional reprogramming. Created with BioRender.com.

Zhou et al. demonstrated that *P. gingivalis* can modulate β-catenin signaling through noncanonical mechanisms in telomerase-immortalized gingival keratinocytes. In these experimental models, infection resulted in gingipain-dependent proteolytic cleavage of β-catenin and GSK-3β, accompanied by degradation of Axin1 and APC, key components of the β-catenin destruction complex [22]. These alterations were associated with increased nuclear accumulation of β-catenin and enhanced TCF/LEF transcriptional activity, effects that were abrogated by pharmacologic inhibition of gingipain activity [38,39]. Notably, β-catenin modulation occurred in the absence of exogenous Wnt ligand stimulation, indicating that *P. gingivalis* can influence β-catenin availability independently of the canonical ligand–receptor axis. While these findings establish a mechanistic link between gingipain activity and β-catenin stabilization under controlled conditions, they derive from simplified epithelial models and do not by themselves establish relevance to human OSCC [22]. These observations instead highlight one potential pathway through which chronic microbial exposure could perturb epithelial signaling in inflamed oral tissues (Figure 2).

From a molecular standpoint, gingipains represent key bacterial proteases capable of directly reshaping host signaling architecture, highlighting proteolytic host–microbe interactions as a central biochemical interface in periodontal dysbiosis.

### 5.2. Chronic Infection, Transformation, and β-Catenin-Related Gene Signatures

Long-term infection models provide additional insight into how sustained *P. gingivalis* exposure may alter epithelial cell behavior over time. In human immortalized oral epithelial cells subjected to continuous infection for 15–23 weeks, progressive acquisition of pro-oncogenic features was observed, including altered morphology, increased proliferation, and enhanced migratory and invasive capacity [40]. These phenotypic changes are consistent with a shift away from normal epithelial homeostasis toward a more plastic and stress-adapted cellular state.

Transcriptomic and proteomic analyses of chronically infected cells revealed widespread reprogramming across multiple signaling pathways involved in cell cycle regulation, apoptosis resistance, DNA replication, and metabolic adaptation [41,42]. Within this broader landscape, components of the Wnt/β-catenin pathway were among several signaling networks found to be dysregulated, and pathway enrichment analyses repeatedly identified β-catenin-associated signaling as one of multiple intersecting hubs affected by prolonged infection [43,44]. Importantly, β-catenin dysregulation occurred alongside alterations in other oncogenic pathways, underscoring that no single pathway fully accounts for the observed cellular phenotype.

The biological relevance of these changes was further explored using xenograft models, in which chronically infected epithelial cells exhibited increased tumor formation compared with uninfected controls [45]. While these findings indicate that sustained microbial exposure can modify tumor-associated properties in experimental systems, interpretation is constrained by the use of immunodeficient hosts and pre-conditioned epithelial cells. As such, these models provide mechanistic insight into pathway perturbation rather than direct evidence of microbial causation in human OSCC.

### 5.3. Effects on Established OSCC Cells

**Figure 3:**
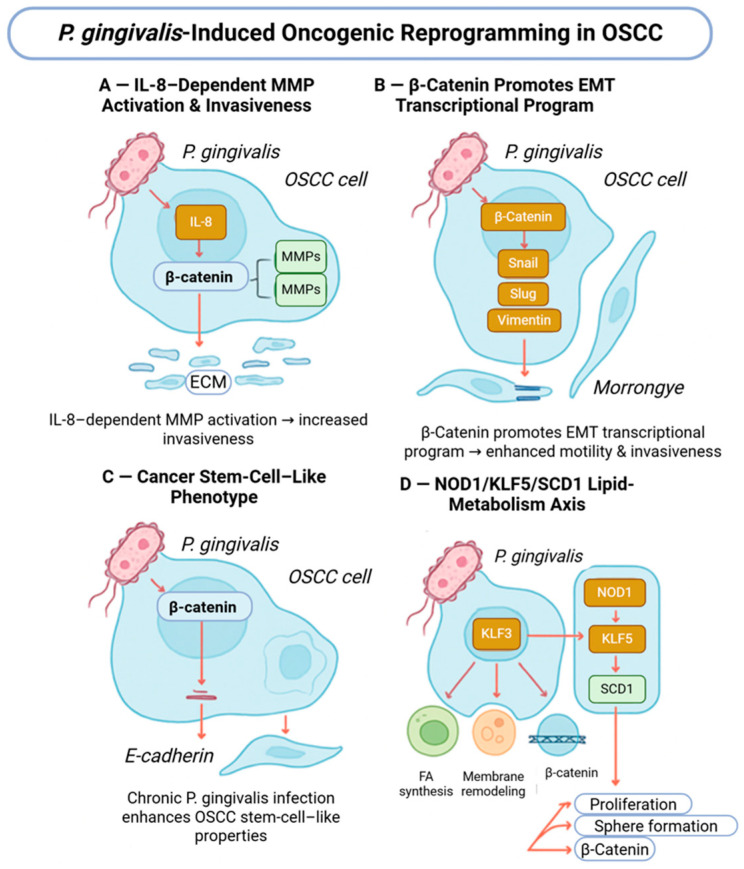
*Porphyromonas gingivalis*-induced oncogenic reprogramming in oral squamous cell carcinoma. (**A**) *P. gingivalis* enhances IL-8-dependent MMP activation, promoting extracellular matrix degradation and tumor invasiveness. (**B**) β-Catenin activation drives EMT-related transcriptional programs, facilitating tumor cell migration. (**C**) Chronic *P. gingivalis* exposure promotes cancer stem cell-like properties through β-catenin signaling. (**D**) Modulation of lipid metabolism via the NOD1/KLF5/SCD1 axis supports proliferation, survival, and aggressive tumor behavior. Created with BioRender.com.

In established OSCC cell lines, *P. gingivalis* exposure has been shown to enhance invasive behavior, modulate inflammatory signaling, and promote epithelial–mesenchymal transition-associated gene expression [46]. These effects are mediated through multiple pathways, including IL-8-dependent matrix metalloproteinase activation and induction of EMT-related transcription factors. β-Catenin signaling intersects with these processes by interacting with EMT and stemness-associated regulatory networks, suggesting cooperative rather than exclusive pathway involvement (Figure 3).

Recent studies further demonstrate that *P. gingivalis* can influence lipid metabolic reprogramming through the NOD1/KLF5/SCD1 axis, which interfaces with β-catenin as well as other oncogenic transcriptional programs [30]. These observations reinforce the concept that *P. gingivalis* affects a spectrum of host signaling pathways in OSCC cells, with β-catenin representing one component of a broader, interconnected response rather than a singular mechanistic driver.

## 6. Mechanistic Integration: How Might *P. gingivalis*–β-Catenin Cross-Talk Promote Oral Carcinogenesis?

### 6.1. Disruption of Epithelial Barrier and E-Cadherin/β-Catenin Complex

**Figure 4:**
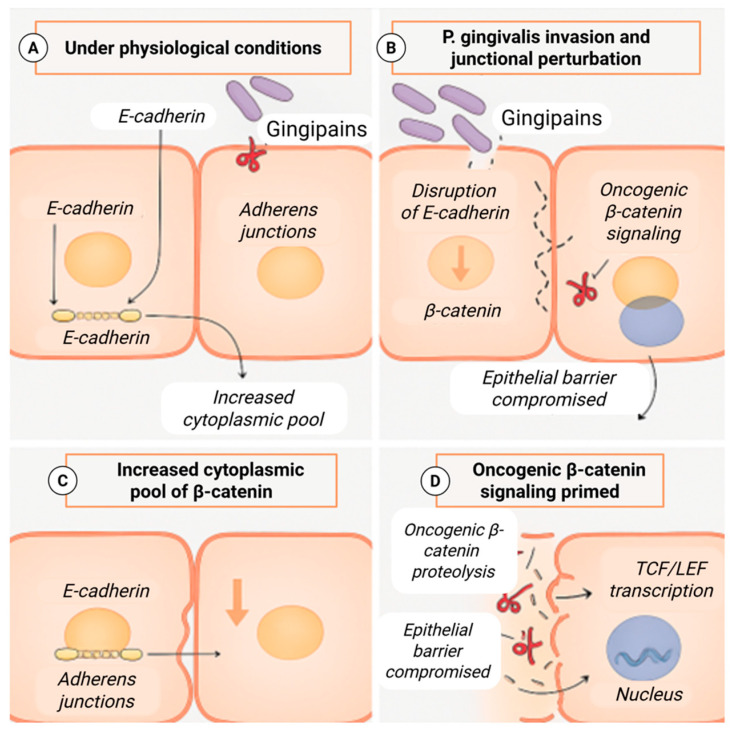
*Porphyromonas gingivalis*-mediated disruption of E-cadherin/β-catenin signaling in oral squamous cell carcinoma. (**A**) Under physiological conditions, E-cadherin sequesters β-catenin at adherens junctions, maintaining epithelial integrity. (**B**) *P. gingivalis* gingipains disrupt E-cadherin, compromising junctional stability and epithelial barrier function. (**C**) Loss of E-cadherin increases the cytoplasmic pool of β-catenin. (**D**) Stabilized β-catenin translocates to the nucleus, activates TCF/LEF-dependent transcription, and drives oncogenic signaling. Created with BioRender.com.

In stratified oral epithelium, adherens junctions play a critical role in maintaining barrier integrity and regulating intracellular signaling. Under physiological conditions, E-cadherin sequesters β-catenin at the plasma membrane, limiting its participation in transcriptional programs. Loss or redistribution of junctional components increases the cytoplasmic availability of β-catenin and alters epithelial polarity and cohesion. Experimental studies indicate that *P. gingivalis* can perturb adherens junction organization and E-cadherin localization in epithelial cells, thereby increasing the pool of β-catenin released from membrane sequestration [47] (Figure 4).

Rather than constituting an oncogenic event in isolation, junctional disruption represents a permissive alteration that may sensitize epithelial cells to additional inflammatory and stress-related signals. In this context, *P. gingivalis*-associated weakening of epithelial barrier function may contribute to altered signaling competence and increased epithelial plasticity without being sufficient to initiate malignant transformation on its own.

### 6.2. Proteolytic Dismantling of the β-Catenin Destruction Complex

**Figure 5:**
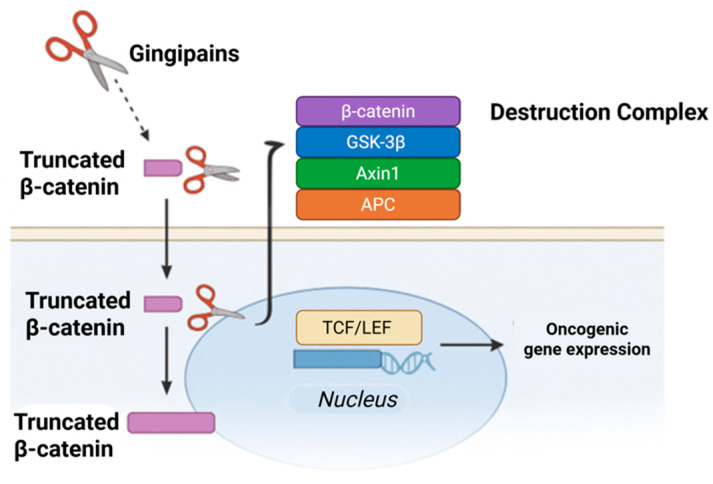
Gingipain-mediated truncation of β-catenin and oncogenic signaling. *Porphyromonas gingivalis* gingipains proteolytically truncate β-catenin, allowing it to evade the destruction complex and translocate to the nucleus, where it engages TCF/LEF transcription factors to induce oncogenic gene expression. Created with BioRender.com.

Proteolytic interference with components of the β-catenin destruction complex represents another experimentally observed mechanism by which *P. gingivalis* can influence intracellular signaling dynamics. Gingipain-mediated cleavage of β-catenin and associated regulatory proteins has been shown to alter β-catenin stability and subcellular localization under controlled conditions [38] (Figure 5). These findings illustrate how microbial proteases can bypass canonical regulatory checkpoints governing β-catenin turnover.

Importantly, such proteolytic events have been demonstrated primarily in simplified epithelial models and should be interpreted as mechanistic possibilities rather than evidence of pathway dominance in human disease. Within a broader signaling context, disruption of β-catenin regulation likely interacts with parallel inflammatory and metabolic pathways rather than acting as an independent determinant of carcinogenesis.

### 6.3. Convergence with Inflammatory and Innate Immune Signaling

**Figure 6:**
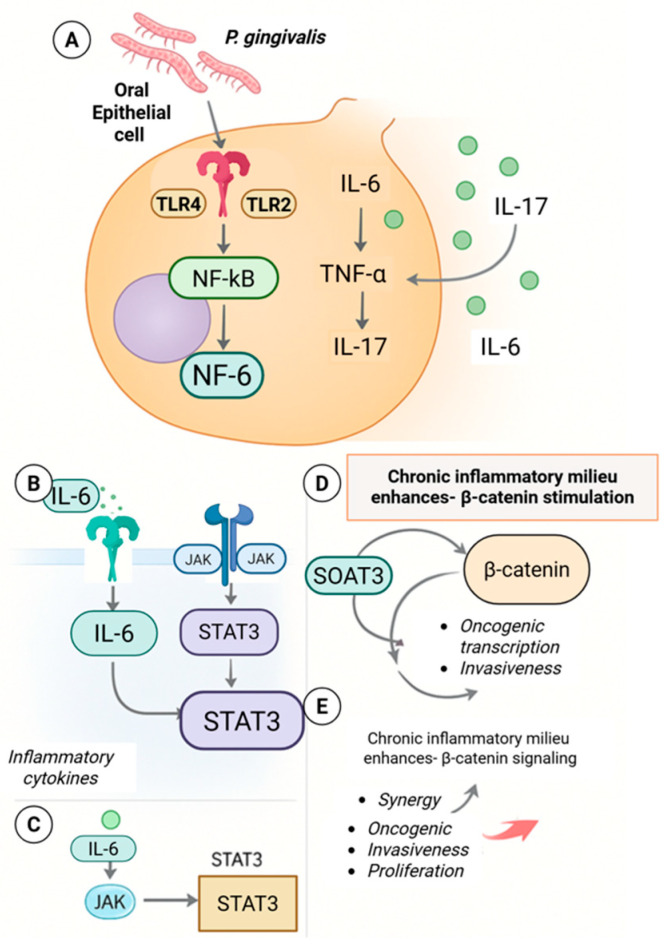
Inflammation-driven β-catenin activation in *Porphyromonas gingivalis*-associated oral carcinogenesis. (**A**) *P. gingivalis* activates TLR2/4–NF-κB signaling in oral epithelial cells, inducing IL-6, IL-17, and TNF-α release. (**B**,**C**) IL-6 activates JAK/STAT3 signaling, amplifying proinflammatory cytokine production. (**D**) Chronic inflammatory signaling enhances β-catenin stabilization and transcriptional activity. (**E**) Crosstalk between STAT3 and β-catenin promotes oncogenic transcription, invasion, and proliferation. Created with BioRender.com.

Chronic exposure to *P. gingivalis* is associated with sustained activation of innate immune signaling pathways, including NF-κB and IL-6–STAT3 signaling, in epithelial and immune cells [48,49]. These inflammatory cascades intersect with β-catenin signaling at multiple levels, including transcriptional cooperation and shared target gene regulation (Figure 6). In experimental systems, inflammatory cytokines can enhance β-catenin transcriptional output, while β-catenin can, in turn, modulate inflammatory gene expression.

This bidirectional interaction suggests that β-catenin functions as part of a broader inflammatory signaling network rather than as a solitary oncogenic pathway. Persistent low-grade inflammation may therefore amplify epithelial plasticity and survival signaling in a context-dependent manner, without implying that β-catenin activation alone drives malignant transformation.

### 6.4. Epithelial Plasticity, Invasion, and Stem-like Phenotypes

β-Catenin signaling intersects with transcriptional programs associated with epithelial–mesenchymal transition, invasion, and cellular plasticity. Experimental studies demonstrate that *P. gingivalis* exposure can enhance EMT-associated gene expression, matrix remodeling, and invasive behavior in epithelial and OSCC cell models [50,51]. These changes occur alongside activation of multiple oncogenic pathways, including inflammatory and metabolic regulators.

**Figure 7:**
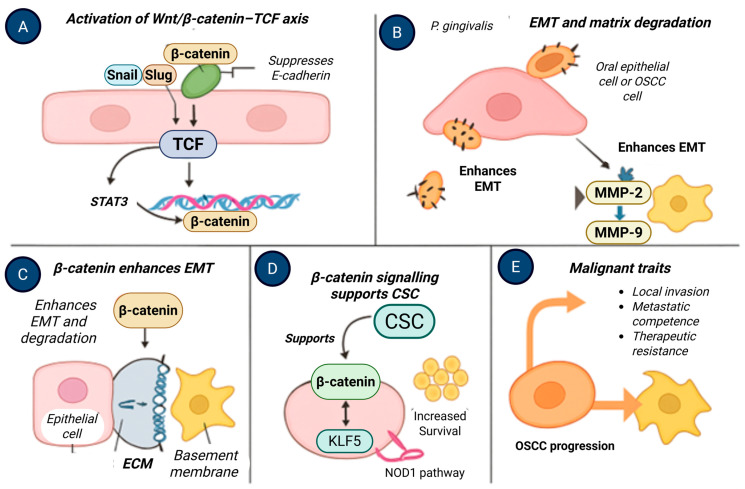
β-Catenin-driven epithelial–mesenchymal transition and malignant progression in oral squamous cell carcinoma. (**A**) Activation of the Wnt/β-catenin–TCF axis induces EMT transcription factors and suppresses E-cadherin. (**B**) *Porphyromonas gingivalis* enhances EMT and matrix degradation through MMP-2 and MMP-9 activation. (**C**) β-Catenin promotes epithelial–mesenchymal transition and extracellular matrix breakdown. (**D**) β-Catenin signaling supports cancer stem cell-like properties via KLF5-dependent pathways. (**E**) These processes collectively drive OSCC progression, invasion, and therapeutic resistance. Created with BioRender.com.

Similarly, enrichment of stem-like cellular phenotypes following prolonged *P. gingivalis* exposure has been reported in experimental systems, with β-catenin acting in concert with additional transcriptional regulators such as KLF5 and NOD1-associated pathways [52,53] (Figure 7). These observations support a model in which β-catenin contributes to epithelial plasticity as part of a coordinated response to chronic microbial and inflammatory stress, rather than functioning as a singular driver of stemness or malignancy.

### 6.5. Immune Modulation and Tumor Microenvironmental Context

Beyond epithelial-intrinsic effects, *P. gingivalis* can influence the tumor microenvironment through modulation of host immune responses. Experimental evidence indicates that this pathogen can persist within macrophages and alter their functional polarization, promoting immunoregulatory phenotypes that may attenuate antitumor surveillance [54]. These immune-restricted effects operate in parallel with epithelial signaling alterations and represent a distinct, non-redundant axis of host–microbe interaction.

**Figure 8:**
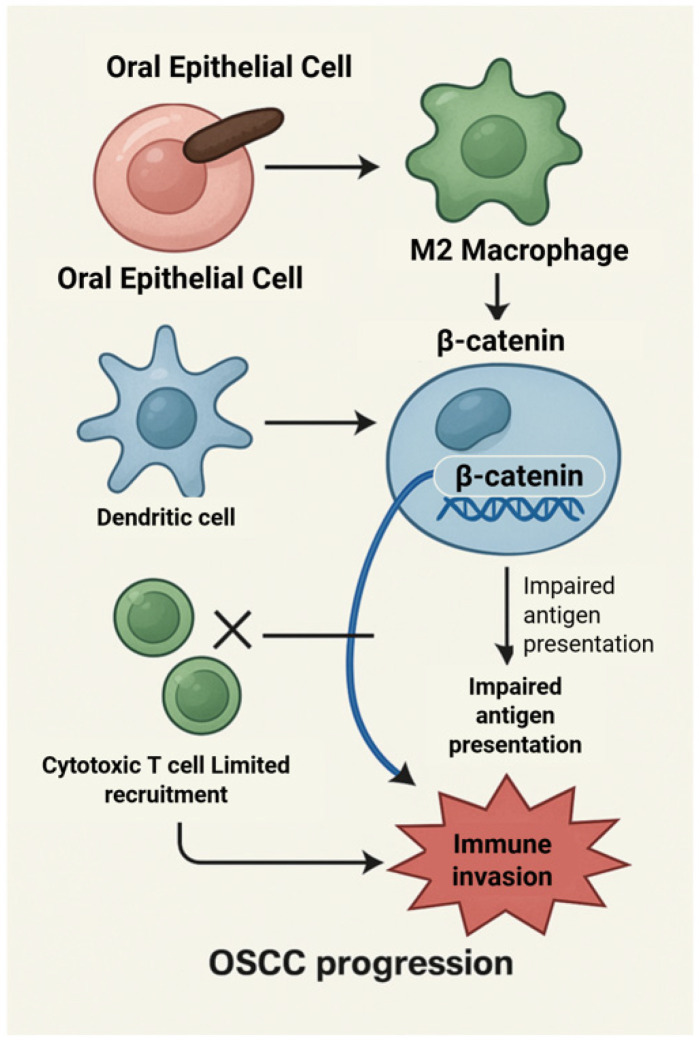
β-Catenin-mediated immune evasion in oral squamous cell carcinoma. β-Catenin activation in oral epithelial and immune cells impairs antigen presentation by macrophages and dendritic cells, limits cytotoxic T-cell recruitment, and promotes immune evasion, thereby facilitating OSCC progression. Created with BioRender.com.

Within immune cells, β-catenin signaling has been implicated in the regulation of antigen presentation, dendritic cell function, and T-cell recruitment [55]. While direct evidence linking *P. gingivalis* to β-catenin activation in immune cells remains limited, its capacity to modulate upstream inflammatory mediators provides a plausible framework for indirect pathway interaction [56,57] (Figure 8). This proposed immune-specific role of β-catenin is speculative and highlights an area requiring targeted investigation.

Collectively, these epithelial and immune-modulatory mechanisms underscore the multifactorial nature of oral carcinogenesis. Rather than acting through a single dominant pathway, *P. gingivalis*-associated β-catenin perturbation appears to operate within a complex network of inflammatory, metabolic, and microenvironmental signals that may modify disease trajectory under specific biological contexts [58,59].

### 6.6. Strain Heterogeneity of Porphyromonas gingivalis and Implications for β-Catenin Modulation

An important microbiological consideration when interpreting the mechanistic role of *P. gingivalis* in epithelial signaling modulation relates to strain-level heterogeneity. *P. gingivalis* exhibits substantial genetic and phenotypic diversity across clinical and laboratory strains, with differences in virulence factor expression, including gingipain protease activity, fimbrial subtype composition (FimA genotypes I–VI), outer membrane vesicle production, and immune-modulatory capacity [60]. These variations can significantly influence host–pathogen interactions, epithelial invasion efficiency, and downstream signaling responses [60]. Experimental studies have demonstrated that gingipains, key cysteine proteases produced by *P. gingivalis*, play a central role in modulating host signaling pathways, including disruption of epithelial junctional complexes and alteration of β-catenin regulatory dynamics [60,61]. However, gingipain expression levels and enzymatic activity vary among strains, potentially affecting the magnitude and nature of host signaling perturbation [61,62]. Similarly, strain-specific differences in fimbrial structure influence bacterial adhesion, invasion, and intracellular persistence, which may indirectly affect epithelial signaling responses, including β-catenin stabilization and transcriptional activation [63,64].

In addition, genomic variability among *P. gingivalis* strains contributes to differences in inflammatory signaling induction, immune evasion, and metabolic adaptation, all of which may interact with epithelial regulatory pathways relevant to carcinogenesis. Most mechanistic investigations have relied on a limited number of laboratory-adapted strains, such as ATCC 33,277 and W83, which may not fully represent the diversity of clinical isolates present in human oral microbiomes [65,66]. These observations suggest that the capacity of *P. gingivalis* to modulate β-catenin signaling and influence epithelial plasticity may vary across strains and host environments. Accordingly, *P. gingivalis* should be regarded as a heterogeneous microbial species with strain-dependent pathogenic potential rather than as a uniform biological entity. Future studies integrating strain-resolved microbial profiling with molecular characterization of host signaling responses will be essential to clarify the microbiological specificity and translational relevance of these mechanistic interactions.

## 7. Discussion

This review critically evaluated epidemiologic and experimental evidence linking periodontal disease, *Porphyromonas gingivalis*, and oral squamous cell carcinoma (OSCC), with particular attention to β-catenin signaling as a potential mechanistic intersection. Taken together, available data support a biologically plausible association between chronic periodontal dysbiosis and OSCC-related molecular alterations, but they do not establish causality. Importantly, the strength and interpretation of this evidence vary substantially across study designs, underscoring the need for careful distinction between association, mechanistic plausibility, and disease causation.

From a population perspective, the potential relevance of periodontal–OSCC interactions must be interpreted within a complex epidemiologic landscape. Global Burden of Disease analyses document parallel increases in both periodontal disease and lip and oral cavity cancers over recent decades, particularly in low- and middle-income regions [67].

Although multiple epidemiologic studies report statistically significant associations between clinical indicators of periodontitis and OSCC risk, the magnitude and consistency of these associations vary considerably across study populations and methodological approaches [68]. Reported effect estimates are generally modest, and associations are often attenuated after adjustment for major confounders, including tobacco use, alcohol consumption, socioeconomic status, and healthcare access. Moreover, periodontal exposure definitions differ substantially across studies, ranging from tooth loss and clinical attachment loss to self-reported periodontal history, further complicating direct comparison and interpretation [69]. This heterogeneity suggests that periodontal disease may function, at least in part, as a surrogate marker of cumulative inflammatory burden, health behaviors, or environmental exposures rather than as an independent causal determinant.

A central limitation of the current epidemiologic evidence relates to temporality. Most available studies are cross-sectional or retrospective in design and assess periodontal status at or near the time of OSCC diagnosis. As a result, it remains difficult to determine whether periodontal dysbiosis precedes carcinogenesis and contributes to tumor initiation, whether it modifies tumor progression, or whether observed microbial and inflammatory changes arise secondarily from tumor-associated ecological disruption [70,71,72]. Tumor-related alterations in oral anatomy, immune function, and local microbial niches may themselves favor colonization by anaerobic periodontal pathogens, including *P. gingivalis*, thereby complicating causal inference. The absence of prospective longitudinal studies integrating quantitative microbial profiling, periodontal assessment, and molecular characterization of premalignant oral lesions represents a major gap in the current evidence base. Addressing temporality through well-designed longitudinal cohorts will be essential to clarify whether periodontal dysbiosis represents a causal contributor, a disease modifier, or an epiphenomenon within the broader context of oral carcinogenesis.

Experimental studies provide mechanistic insight into how *P. gingivalis* can perturb epithelial signaling under controlled conditions. In vitro and animal models demonstrate that this pathogen can influence β-catenin availability, transcriptional activity, and downstream cellular phenotypes relevant to proliferation, epithelial plasticity, and metabolic adaptation [22,30,39,73]. These findings establish biological plausibility but must be interpreted within the constraints of simplified experimental systems, including immortalized cell lines, immunodeficient hosts, and chemical carcinogenesis models. Consequently, mechanistic data should be regarded as hypothesis-generating rather than confirmatory of pathogenic relevance in human OSCC.

Oral carcinogenesis occurs within the context of a complex and dynamic microbial ecosystem rather than in response to a single pathogenic organism. Increasing evidence indicates that OSCC-associated microbial communities are characterized by polymicrobial dysbiosis involving multiple anaerobic taxa, including *Fusobacterium nucleatum* (*F. nucleatum*), *Prevotella intermedia* (*P. intermedia*), *Treponema denticola* (*T. denticola*), and other periodontitis-associated species [74,75]. These organisms may interact synergistically through cooperative metabolic activity, inflammatory amplification, and ecological niche modification, collectively influencing epithelial signaling pathways and tumor-associated microenvironmental conditions. In this framework, *P. gingivalis* should be regarded not as an isolated causal agent but as one component of a broader dysbiotic microbial consortium capable of modulating host epithelial biology [21,76]. The mechanistic focus on *P. gingivalis* in this review reflects the availability of experimentally characterized host–pathogen signaling interactions rather than implying exclusivity or primacy in OSCC pathogenesis.

Crucially, β-catenin does not operate in isolation. Experimental evidence indicates that *P. gingivalis* modulates a broad spectrum of host pathways, including inflammatory, metabolic, stress-response, and immune-regulatory networks [77]. β-Catenin intersects with these pathways through transcriptional cooperation, shared regulatory targets, and microenvironmental feedback loops. This convergence suggests that β-catenin functions as an integrative signaling node within a broader oncogenic network rather than as a dominant or universal driver of malignant transformation. Such a network-based interpretation aligns with the molecular heterogeneity of OSCC and helps explain variability across experimental and clinical observations.

Despite mechanistic consistency across experimental platforms, substantial knowledge gaps remain. Longitudinal human studies capable of defining temporal relationships between *P. gingivalis* exposure, β-catenin dysregulation in premalignant lesions, and OSCC progression are notably lacking. Most clinical studies rely on cross-sectional sampling and do not integrate quantitative microbial profiling with molecular assessment of β-catenin signaling within oral potentially malignant disorders [27,44,78]. Additionally, strain-level heterogeneity in *P. gingivalis* virulence traits and host susceptibility factors are rarely addressed, further complicating translational interpretation [29].

Within these limitations, *P. gingivalis* may be cautiously regarded as a biologically plausible microbial risk modifier rather than a causal agent of OSCC [79]. Unlike genetic alterations, microbial exposures are potentially modifiable, raising interest in whether periodontal therapy, antimicrobial strategies, or microbiome modulation could indirectly influence epithelial signaling environments associated with oral carcinogenesis [80,81,82]. However, such translational implications remain speculative and require validation through well-designed interventional and biomarker-driven studies.

Several limitations of the current evidence base warrant emphasis. Much of the available mechanistic insight derives from in vitro systems, immortalized epithelial models, and chemically induced animal carcinogenesis, which incompletely recapitulate the complexity of human oral squamous cell carcinoma. Epidemiologic studies remain largely observational and are subject to residual confounding from tobacco exposure, alcohol consumption, socioeconomic status, and access to oral healthcare, while strain-level heterogeneity in *Porphyromonas gingivalis* virulence and interindividual host susceptibility are rarely addressed. Moreover, longitudinal human studies integrating quantitative microbial profiling with molecular assessment of premalignant oral lesions are notably lacking, limiting inference regarding temporal relationships. Within these constraints, the conceptual contribution of this review lies in integrating dispersed experimental findings within a signaling-network framework that situates β-catenin at the interface of microbial, inflammatory, and metabolic pathways relevant to oral epithelial biology. Rather than proposing a single mechanistic axis, this perspective highlights how chronic microbial stress may reshape epithelial signaling landscapes in a context-dependent manner. Future research should therefore prioritize prospective cohort studies and mechanistically informed interventions to clarify whether modulation of periodontal dysbiosis translates into meaningful changes in molecular risk profiles or clinical outcomes in OSCC.

## 8. Clinical Implications

### 8.1. Periodontal Status and OSCC Risk Stratification

From a clinical perspective, the mechanistic and epidemiologic evidence reviewed here raises the possibility that severe, untreated periodontitis—particularly when associated with sustained *P. gingivalis* colonization—may act as a contextual modifier of OSCC risk rather than an independent determinant [83]. At present, definitive risk estimates are lacking, and periodontal disease should not be regarded as a stand-alone risk factor for OSCC. Nevertheless, these findings suggest that periodontal status may represent a component of the broader inflammatory and microbial milieu that influences oral epithelial biology.

In this context, incorporation of periodontal assessment into OSCC risk evaluation frameworks remains exploratory. Such approaches may be most relevant in carefully selected populations, including individuals without traditional behavioral risk factors but with chronic inflammatory oral conditions. Importantly, any integration of periodontal parameters into screening or risk stratification strategies would require validation through prospective, population-based studies before clinical implementation.

### 8.2. Biomarker Development Considerations

Experimental and clinical observations reviewed herein suggest that microbial and host signaling markers—including *P. gingivalis* DNA, pathogen-specific antibody responses, and indicators of β-catenin pathway activity—may warrant investigation as candidate biomarkers in oral potentially malignant disorders [84]. In principle, combining microbial exposure markers with molecular signatures of epithelial signaling could enhance biological specificity beyond single-parameter assays.

However, existing data are insufficient to support clinical use of such markers. Most studies are cross-sectional, heterogeneous in methodology, and lack standardized thresholds or longitudinal outcome correlation. Salivary, plaque-based, or tissue-based assays quantifying *P. gingivalis* burden and β-catenin-related transcripts therefore remain investigational and should be considered hypothesis-generating tools pending validation in large, well-characterized prospective cohorts.

### 8.3. Therapeutic and Preventive Perspectives

The concept that microbial modulation of host signaling pathways may influence OSCC biology raises interest in potential preventive or adjunctive therapeutic strategies. At present, these concepts remain largely preclinical and speculative. Approaches discussed in the literature include intensified periodontal therapy aimed at reducing chronic microbial burden [85], pharmacologic inhibition of gingipain activity [86], and modulation of Wnt/β-catenin signaling in selected experimental contexts [87]. In parallel, microbiome-targeted strategies—such as probiotics, bacteriophage-based approaches, or targeted delivery systems—have been proposed as methods to selectively alter pathogenic microbial communities [88].

Crucially, none of these strategies has been demonstrated to reduce OSCC incidence, progression, or recurrence in humans. Potential risks, off-target effects, and unintended disruption of host–microbiome homeostasis remain significant concerns. Accordingly, these approaches should be viewed as areas for future investigation rather than as actionable clinical interventions.

These observations support future exploration of periodontal biomarkers and microbial modulation as adjunctive components of OSCC risk stratification rather than independent screening tools.

## 9. Conclusions

Global Burden of Disease 2019 analyses underscore that both periodontal disease and oral squamous cell carcinoma (OSCC) represent high-burden conditions with increasing prevalence and substantial disability-adjusted life year impact, particularly in low- and middle-income regions. Within this epidemiologic context, accumulating experimental evidence supports a biologically plausible association between chronic *Porphyromonas gingivalis* exposure and molecular alterations relevant to OSCC biology, including dysregulation of β-catenin signaling. In vitro and in vivo studies demonstrate that *P. gingivalis* can influence β-catenin availability and transcriptional activity through multiple context-dependent mechanisms, including junctional disruption, proteolytic interference with regulatory complexes, and interaction with inflammatory, autophagic, and metabolic pathways.

Despite this mechanistic coherence, definitive evidence establishing a causal role for *P. gingivalis* in human OSCC development remains lacking. Epidemiologic associations are heterogeneous and subject to residual confounding, and experimental models do not fully recapitulate the complexity of human disease. Accordingly, chronic *P. gingivalis*-associated periodontitis should be regarded cautiously as a potential biological modifier of oral epithelial signaling environments rather than as an independent etiologic factor in OSCC pathogenesis.

The conceptual value of recognizing a *P. gingivalis*–β-catenin interface lies in its ability to frame testable hypotheses at the intersection of microbial dysbiosis, chronic inflammation, and epithelial signaling plasticity. This perspective highlights important opportunities for future research, including longitudinal human studies integrating quantitative microbial profiling with molecular characterization of premalignant lesions, and mechanistic investigations designed to clarify the relative contribution of β-catenin signaling within broader oncogenic networks. Addressing these questions will be essential for determining whether modulation of periodontal dysbiosis or associated signaling pathways has relevance for OSCC risk assessment or prevention within global oral health and cancer control strategies.

From a molecular perspective, these observations emphasize protease-mediated signaling modulation and β-catenin network plasticity as candidate biochemical interfaces linking periodontal dysbiosis to epithelial stress adaptation.

## Figures and Tables

**Table 1:** Integrated Evidence Linking *P. gingivalis* to Oral Squamous Cell Carcinoma Across Study Types.

Study Type	Representative Findings	Key Conclusions	Strengths	Limitations
Epidemiological Studies (Case–Control & Cohort) [24,25,26]	Periodontitis indicators (attachment loss, bone loss, tooth loss) are associated with increased OSCC risk after adjustment for smoking and alcohol, with variability across populations.	Periodontal disease and oral dysbiosis are associated with OSCC risk, potentially reflecting shared inflammatory and behavioral determinants.	Large populations; multivariable adjustment for major confounders; reproducible associations across studies.	Observational design; residual confounding likely; periodontal status often assessed cross-sectionally; causality cannot be inferred.
Serologic Studies (Antibody Titers) [27]	Higher *P. gingivalis*-specific antibody levels detected in OSCC patients compared with controls, with population-level heterogeneity.	Indicates systemic immune recognition consistent with chronic exposure to *P. gingivalis* in OSCC patients.	Minimally invasive; quantifiable biomarkers; reflects long-term microbial exposure.	Antibody titers reflect exposure rather than active infection; assay variability across studies.
Microbiological/Molecular Detection (PCR, qPCR, Sequencing) [27]	*P. gingivalis* DNA detected more frequently in saliva, tumor-adjacent epithelium, and resected OSCC tissues compared with controls.	Supports enrichment of *P. gingivalis* within the OSCC-associated oral microenvironment.	High sensitivity; enables localization of microbial signatures in tumor-associated sites.	DNA detection does not confirm bacterial viability, metabolic activity, or functional involvement.
Histopathological Localization (ISH, Immunolabeling) [28]	*P. gingivalis* localized at the tumor invasive front; co-localization with *Fusobacterium nucleatum* suggests polymicrobial presence.	Demonstrates spatial proximity of periodontal pathogens to malignant epithelium.	Tissue-level visualization; precise spatial resolution.	Spatial association alone does not establish mechanistic involvement or causality.
In Vivo Carcinogenesis Models (4NQO Mouse Model) [29,30,31]	Chronic oral infection with *P. gingivalis* ± *F. nucleatum* increases tumor burden, accelerates dysplasia, and enhances invasiveness compared with carcinogen exposure alone.	Provides relatively strong experimental evidence under carcinogen-exposed conditions.	Controlled experimental design; temporal exposure; mechanistic insight.	Animal model with chemical carcinogen; limited generalizability to human OSCC and natural microbial ecology.
Mechanistic Cellular Studies (In Vitro) [32,33]	*P. gingivalis* exposure modulates β-catenin, PI3K/AKT, and STAT3 signaling and induces EMT-associated, anti-apoptotic, and inflammatory responses.	Demonstrates modulation of oncogenic signaling pathways in epithelial cells under experimental conditions.	Mechanistic resolution at pathway level; reproducible and controllable systems.	In vitro models oversimplify tumor microenvironment and host–microbe interactions.

## Data Availability

No new data were generated or analyzed in support of this research. All information discussed in this study is derived from previously published studies cited within the manuscript.
